# Internationally recruited nurses from India and the Philippines in the United Kingdom: the decision to emigrate

**DOI:** 10.1186/1478-4491-7-37

**Published:** 2009-04-24

**Authors:** Álvaro Alonso-Garbayo, Jill Maben

**Affiliations:** 1 London School of Hygiene and Tropical Medicine, London, UK; 2 King's College London, National Nursing Research Unit, London, UK

## Abstract

**Background:**

The United Kingdom has recruited nurses from countries with a reported surplus in their nursing workforce, such as India and the Philippines. However, little is known about the decision to emigrate made by nurses from these countries. One theory suggests that individuals weigh the benefits and costs of migration: the push and pull factors. This paper challenges the restricted economic focus of this predominant theory and compares the diverse motivations of nurses from different countries as well as those of nurses with previous migratory experience and first-time migrants.

**Methods:**

This research was undertaken in a National Health Service acute trust in London by means of a qualitative interpretative approach. Data were collected through face-to-face longitudinal and cross-sectional interviews with internationally recruited nurses from India (n = 6) and the Philippines (n = 15); and analysis of their narratives was used to generate data about their expectations and experiences. Data were analysed by means of a framework approach that allowed for intra-case and cross-case analysis.

**Results:**

From an individual perspective, nurses in this study reported economic reasons as the main trigger for migration in the first instance. Yet this doesn't entirely explain the decision to move from previous migratory destinations (e.g. Saudi Arabia) where economic needs are already fulfilled. In these cases migration is influenced by professional and social aspirations that highlight the influence of the cultural environment – specifically some religious and gender-related issues. Family support and support from migratory networks in the country of origin and destination were also important elements conducive to and supportive of migration. Nurses from India report coming to the United Kingdom to stay, while Filipina nurses come as temporary migrants sending remittances to support their families in the Philippines.

**Conclusion:**

This study shows the diverse motivations of nurses from different countries and with different migratory backgrounds and provides evidence that factors other than economic factors influence nurses' decision to emigrate. This information can help developing countries increase retention of this essential and often scarce resource and can also help the United Kingdom's National Health Service to improve the experience of internationally recruited nurses and therefore increase their retention in the United Kingdom.

## Background

A global shortage of nurses now affects both developed and developing countries [1-3]. In more affluent countries, however, international recruitment is used to address the problem, attracting nurses from low-income countries [4]. The United Kingdom issued a code of conduct for ethical recruitment of overseas nurses in 2001 aimed at protecting health systems in developing countries from the brain drain triggered by active international recruitment [5]. The Department of Health in 2004 issued a list of developing countries that should not be targeted for international recruitment. However, the code of conduct does not cover the private sector and also cannot stop nurses from developing countries independently moving to the United Kingdom and seeking employment [6].

As a result, 10 sub-Saharan African countries included in the list were among the top 25 overseas source countries from which around 300 nurses and midwives were admitted to the Nursing and Midwifery Council (NMC) Register from April 2006 to 31 March 2007 [7]. India and the Philippines have a reported surplus in the production of nurses [8-10]. Both countries have signed agreements with the United Kingdom Department of Health to facilitate nurse emigration [11]. Indian and Filipino nurses often find it difficult to get their first job in their own countries after graduation. There is evidence that nurses decide to undertake nursing studies as a life-improvement strategy via migration and sometimes as a survival strategy [6,12]. Increasing the retention of nurses from countries such as India and the Philippines in the United Kingdom is important to minimize the need of the National Health Service (NHS) to recruit nurses from other countries with acute shortages, such as those in sub-Saharan Africa [1,13].

There is some evidence to suggest that internationally recruited nurses do not stay in their first destination country for long [14,15]. For example, they often use the United Kingdom as a step towards other destinations [14]. Expectations developed during the pre-departure period and during the first weeks after departure, define the basis of a psychological contract between employee and employer [16-18]. The psychological contract is a "sophisticated set of expectations and rules which forms the psychological basis for the continuing commitment of employees to their employer" [18]. Understanding the reasons for nurses to emigrate, from their own perspective, is essential to identifying aspects of that contract and to ensuring that nurses meet their expectations, leading to improved job satisfaction, which in turn is known to increase retention [19].

The decision to emigrate is essentially a personal one [20] resulting from individuals' weighing the benefits and the cost of migration. In order to understand this trade-off, the study reported in this paper draws upon the push-and-pull factors theory, proposed by Ravenstein in the late 1800s [21] and reintroduced by Lee in 1966 [22]. This theory is still commonly used to explain migratory movements of health workers today [12,23-31], which attests to its flexibility and clarity.

Push factors are those forces in countries of origin that impel workers to emigrate. Pull factors are those from destination countries that attract professionals [12,32]. Pull and push factors are commonly opposite aspects of similar phenomena in source and destination countries [12,33].

Buchan et al. (2003) argued that the extent of the gap between both sides determines the strength of the pulling influence from destination countries [34]. It is argued that push and pull factors determine the flow direction by attracting or repelling health workers. Other factors such as professional regulations (registration and licensing) and migration and labour policies in source and destination countries modulate the size of the flow [35].

Theories about migration have been used to explain movements among specific professional groups, but the study of nurse migration is a relatively new area. Although limited, there is an incipient body of literature about nurses' motivations for migration. Most scholars agree that they relate to professional, economic, social and personal reasons [4,12,24,28,30,36-39]. Push and pull factor theory constituted the theoretical background for most of these studies.

At an economic level, most studies found that on the pull side, economic improvements, employment availability, ensuring a good retirement pension and expectations to improve quality of life were the main reasons for nurses to emigrate [24,30,31,40,41]. With regard to employment availability, some authors argue that the shortages of nurses in developed countries and their active international recruitment constitute an important pull factor for nurses from developing countries [20,31,42].

Nurses are often seen as exclusively economic migrants, but studies suggest that this represents a limited understanding of nurses' motives [29], which include professional motives as a very strong incentive for migration, sometimes outweighing economic factors [38]. From a professional perspective, issues related to professional development, such as access to continuing education, work experience or working within relatively higher nursing health care standards were common findings in many of the studies [24,40,43]. Less-experienced nurses leave their countries looking for opportunities to apply their recently acquired knowledge and skills, while senior nurses leave in search of a better professional career [36].

Other professional factors relate to the work environment, such as access to better technology, availability of clinical resources, improved management or professional autonomy [30,37]. Nurses also expect to have greater responsibility in practice and to undertake relatively more complex tasks than in their countries of origin [40].

Other factors pushing nurses to leave their countries are conflict, insecurity or political instability. They seek asylum in safer countries, using their nursing skills to find employment and settle [12,25,31].

Old colonial ties to specific countries have also been identified as an important influence on migration and choice of destination. Ranghuram (2009) argues that analysis of health professionals' migration should include a post-colonial perspective, particularly in regard to the United Kingdom's health system [44]. In the same vein, McNeil-Walsh (2004) explains the post-colonial influence on the decision by South African nurses to move to the United Kingdom [45].

Arango (2000) argues that to analyse migration by means of theories that explain only why people move is a limited approach. There are people in countries of origin living in the same conditions as migrants who decide not to move. He proposes that broadening the focus from individual to societal perspectives of migration, including the social costs of adaptation to the new environment, is needed [46].

Thus nurses' decision to emigrate is complex and is likely to be influenced by factors beyond the purely economic. This study takes a broad perspective, examining factors in addition to the economic and professional aspects involved in this important decision – those of a social and cultural nature.

## Methods

Data on the decision to emigrate were collected and analysed between 2005 and 2007 as part of a doctoral thesis [47]. The research is designed as a case study undertaken in an NHS acute Trust in London. London was selected because the proportion of international nurses is greater than anywhere else in the United Kingdom [25]. The specific Trust was selected essentially because it had a history of international recruitment over four years, was actively recruiting overseas nurses at the time of data collection and was willing to participate in the research and allow access. The research uses a qualitative (interpretive) approach with internationally-recruited nurses (IRNs) from India and the Philippines, using analysis of their narratives to generate data about their expectations and experiences [48].

There are two main elements in this research. The first is a longitudinal study of six Indian nurses, who were interviewed three times over eight months from the date of arrival in the United Kingdom in 2005, and 10 of their managers and mentors. The second element comprised Filipina nurses recruited from two cohorts; six nurses who had been in post in the United Kingdom for 18 months and nine nurses recruited by the Trust four years previously.

The main method for data collection was face-to-face, individual, semi-structured interviews. Data obtained through interviews were analysed by means of a framework approach that helped in obtaining policy-oriented results [49] and in order to keep the integrity of the accounts of individual nurses.

The analysis also allowed intra-case and cross-case comparisons. Intra-case comparison was used to contrast the reported experiences of individual Indian nurses at different stages of their migratory experience and adaptation. Cross-case analysis was undertaken by contrasting nurses with different lengths of experiences in the United Kingdom; comparing participants with previous migratory experience with others coming to the United Kingdom as their first experience of migration; and also comparing Indian with Filipina nurses.

All interviews were recorded and transcribed verbatim. Analysis of the interview data allowed for inductive analysis and identification of new themes. All data sets were checked against these initial and new emerging themes. This process was repeated until no new themes were found. Themes and categories were then interpreted and mapped, looking for relationships and associations between concepts and typologies derived from them.

In order to enhance the rigour of the study, some criteria proposed by Green and Thorogood (2004) were used [49]. Following their framework, the research was checked for transparency, validity, reliability, reflexivity and comparability. A clear presentation of the methods used and the process followed contributed to enhancing its transparency. To ensure validity of the results, data were initially analysed by the first author (AA-G) and then shared for "peer debriefing" with other scholars, including the co-author of this paper (JM) for discussion of emerging themes [50].

Preliminary results were presented in several academic and professional forums, where feedback was obtained and used to confirm the validity of findings. The use of specific interview protocols for each group contributed to improving reliability. Interview protocols were pilot-tested with Indian and Filipino health professionals, respectively, before starting the data collection, to ensure cultural appropriateness [51].

The inclusion of direct quotes in the presentation of the results allows the reader to read the raw data and the author's interpretations contributing to rigour. Reflexivity refers to the sensitivity about the extent to which the research process and the authors' assumptions and experiences have shaped the data collected and their interpretation [52].

One of the authors (AA-G) has experience working in developing countries; he also had recently gone through the process of adapting to the United Kingdom's health care context as a nurse, which could have led him to play an advocate's role for the nurses. However, being knowledgeable about the topic being investigated is one of the senses that the qualitative researcher should have [53]. The fact that the researcher who collected the data (AA-G) has a nursing background may have helped participants to speak more openly from a technical perspective. It is likely that this also facilitated a stronger bond between researcher and participants. However, the researchers' own education, how they define and conceptualize nursing and their professional experiences may have also affected the understanding of the participants' views in this regard. By being aware of this and trying to keep the right balance between the researchers' (outsiders) and the nurses' (insiders) perspectives, accounts were analysed with these influences in mind.

Green and Thorogood (2004) suggest that comparison is what drives qualitative analysis. In this study, comparison between cases has been the essence of the analysis and has allowed the researcher to hypothesize and theorize about the experiences of overseas nurses coming to work in the United Kingdom [49]. Emerging theory was contrasted against the whole data set, contributing to the rigour of the study. Finally, the comparison of results with empirical findings from other researchers working in the same subject has contributed to validating their theories and to providing consistency to the research findings.

Ethical clearance for this research was obtained from the relevant NHS Research Ethics Committee and from the Ethics Committee of the London School of Hygiene and Tropical Medicine. Confidentiality and anonymity in the process of data collection and analysis and reporting of findings was ensured and participants were thus able to speak freely and openly to the researcher.

## Results

The decision to emigrate is complex and is influenced by and in turn affects multiple spheres of the migrant's life. Three areas arising from the analysis comprised reasons for migration of an individual, social and cultural nature. At the individual level, nurses reported economic and professional reasons, validating previous literature. The social perspective is illustrated by the influence that family and other social networks had on the decision to emigrate. The cultural perspective is explored through the movement of nurses previously working in Saudi Arabia, representing an example of the influence of the cultural context on the decision to move again.

### Individual perspective

When exploring the factors that influenced these nurses to take the decision to emigrate, we need to differentiate between first and subsequent migratory movements. Half the nurses in this study were already living and working outside their home country when they decided to migrate to the United Kingdom. If we concentrate on the first movement to, for example, the Arab states of The Gulf (e.g. Bahrain, Oman or Saudi Arabia) either from the Philippines or India, one of the main push factors expressed by nurses during interviews was low salaries, with the main pull factor being mostly but not exclusively the relatively higher salary in the Arab Gulf States.

Similarly, nurses coming directly to London from India or the Philippines were more likely to express the economic motive more strongly than others, but it was not their only motivation.

"If I go abroad I can earn more and I can do something for my parents and I can bring my family over here and children can get good education" (ID05 Indian nurse who had arrived in the United Kingdom two days before and with no previous overseas experience).

But in the group of nurses for whom the United Kingdom was not the first migratory destination, relatively higher salaries were not expressed as emphatically as by those in their initial migratory movement. Professional and personal factors were, in this case, relatively more influential:

"When I came from Saudi Arabia, I was mostly expecting something that I can gain, like skills and knowledge" (ID28 Indian nurse with more than four years' experience in the United Kingdom and previous overseas experience assigned as a mentor to one of the Indian nurses).

Nurses in India are obliged to work as interns on a voluntary basis for a period of time after graduation. In India as well as in the Philippines, unemployment among nurses is high; it takes some years for recently graduated nurses to find their first remunerated job. This constitutes a push factor for migration, while the availability of employment overseas represents a pull element.

Most Filipina nurses came to the United Kingdom with economic targets. Some expressed their intention to stay until they reached such targets, after which they were planning to go back to the Philippines:

"I want to start a business back home as soon as I have enough money, and as far as I have gained enough experience here. I don't think I am going to stay here for long, I still really miss back home...." (ID20 Filipina nurse with more than four years' experience in the United Kingdom and no previous overseas experience).

Similarly, other Filipina nurses spoke about their plans of starting a business back in the Philippines in different sectors such as farming, the tourist industry or trade. While Filipina nurses reported sending money home to support their families, Indian nurses expressed a different attitude, with far fewer sending remittances home. Most of the Indian nurses said that if after several years they were happy in the United Kingdom, they would stay:

"If it is okay, then I will stay forever, till my retirement, I will stay. If I can bring my family, then I will stay here" (ID07 Indian nurse with seven months' experience in the United Kingdom, with previous overseas experience).

From a professional perspective, lack of opportunities for development in the country of origin and in previous migratory destinations often constituted a major push factor. Nurses frequently know before coming to the United Kingdom that opportunities for professional advancement are inherent in the British professional nursing career system, which is perceived as a strong pull factor.

"I knew before coming that much research is being done here and that there are better opportunities for my career, I mean if you want to study they will allow you to do that" (ID06 Indian nurse who had arrived in the United Kingdom two days before, with previous overseas experience).

Lack of clinical resources or poorly equipped facilities in the country of origin were also identified as push factors by some of the nurses. Having access to more advanced technology and clinical resources was broadly mentioned as an attraction by all groups interviewed. This was more common among nurses coming from public hospitals in India or the Philippines. But perceptions of advanced technology in the United Kingdom did not always meet their expectations, particularly among those coming from the private sector in their countries of origin or from health services in the Arab Gulf States, where equipment was often better and more technologically advanced than in the United Kingdom.

"...the equipment there [in Saudi Arabia] was more new than here... so when I arrived in London it was quite different, I said Oh! This is quite obsolete... In Saudi Arabia they have the latest model...." (ID011 Filipina nurse with more than 15 months' experience in the United Kingdom and previous overseas experience).

Nurses also suggested that by coming to the United Kingdom they expected to improve professionally by practising in an environment with higher standards of care.

"...In UK will be better, both professionally and personally, better professional standards" (ID02 Indian nurse who had arrived in the United Kingdom one day before, with previous overseas experience).

In terms of the psychological contract, an important element in the process of development of expectations among nurses in this study was the recruitment process. The first contact with the employer and the information received during recruitment is essential in the development of expectations and promises that lead to a psychological contract with the employer [18]. Information received during recruitment was often perceived as deficient, not accurate and sometimes misleading:

"When we had our interview we were told that it was 37.5 hrs per week, but we were never informed that it was 12 hours shifts.... From India we were told that we would get so much salary but after coming here we came to know it is nothing... About the hospital they were giving no information, because they were giving us the website to look up at the hospital. [The web site only shows how the hospital will look 10 years hence when its refurbishment will be completed]" (ID05 Indian nurse who had arrived in United Kingdom two months before the interview and with no previous overseas experience).

Trust managers involved in the in-country selection of nurses perceived the performance of the local recruitment agency in providing information as unsatisfactory:

"In theory they would have been briefed by the agency just before we started interviewing, and actually during the course of the first morning it was becoming obvious that people's ideas of where they were coming to, was really not particularly accurate... that's what they had been told and actually the agency had glossy pictures of Buckingham Palace and Tower Bridge and the Houses of Parliament and that's what they were given to look at during the briefing session" (ID/Recr.1 Trust Nurse manager involved in the recruitment of nurses in India).

As presented above, nurses decided to emigrate pushed by individual motives of an economic and professional nature sometimes reinforced by the information received during their recruitment. However, they also spoke about how the social environment in which they were living either in their home countries or in previous migratory destinations influenced that decision.

### The social perspective

Many of the nurses interviewed, regardless of their nationality or previous migratory experience had followed other colleagues and friends.

"...Because I had friends from the Philippines in Saudi Arabia so that encouraged me to go there because at least I had friends willing to give a hand in case of a crisis..." (ID13 Filipina nurse with 16 months' experience in the United Kingdom and with previous overseas experience).

All the nurses in this study were recruited in groups. These groups, often called "batches" by Filipina nurses, provided an important social network that supported them, particularly during the early stages of the process of adaptation to the United Kingdom.

Family members are often influential actors in the decision-making process. Among the Filipina nurses in this study, the mother was sometimes mentioned as important, not only in the decision to emigrate but often also in the decision to undertake nursing studies. One of the nurses suggested that her mother, moved by the experience of another daughter who became a nurse and was already working abroad, pushed her to undertake nursing studies against her own preferences.

"I didn't have the plan to be in nursing. What I wanted to be is an engineer but my mother told me it was better to take nursing like my sister...." (ID09 Filipina nurse with 15 months' experience in the United Kingdom and previous overseas experience).

Another factor of a social nature, which contributed to the decision to emigrate identified by some nurses, was the higher social status and increased social respect assigned to migrant nurses back in India and in the Philippines.

"Oh I had to go abroad, and then when I go back everybody will have that feeling that I am coming from abroad and all will respect me" (ID05 Indian nurse who had arrived in the United Kingdom two days before, with no previous overseas experience).

Nurses often expected to grow personally through the experience of migration. Living and working in a multicultural environment was perceived as contributing to that experience, which was important for them. The importance of the cultural environment in which nurses were living and how that contributed to their decision to emigrate is illustrated in the next section.

### The cultural perspective

Other factors that contributed to the decision to emigrate were of a cultural nature, such as religion or factors related to gender. In particular, nurses who had been working in Saudi Arabia mentioned that one reason for their move to the United Kingdom was to be able to practise their religion freely; being a group that expressed strong Roman Catholic convictions, this was perceived as an important push factor.

"Religion wise, we were very restricted. During worship we couldn't let the sound go outside. We had one room separated for that. We sealed the room, and kept that on Sundays for all of our friends to come together and have the prayers" (ID07 Indian nurse with 1.5 months' experience in the United Kingdom and previous overseas experience).

Many also expressed their disappointment that being women living within an Islamic society was difficult due to perceived restrictions, such as their choice of dress or restrictions in their movements.

"As a woman you have to cover your face and you can only move within your accommodation [nurses' residential premises], you can't go out, you can only go out with the hospital's bus. Women there are not free" (ID10 Filipino nurse with 15 months' experience in the United Kingdom and previous overseas experience).

Gender in this study has been considered more from a cultural than a social perspective, as cultural aspects of both migrant groups are strong in influencing this aspect. However, the authors acknowledge the broadness of the term and the potential to be considered from both perspectives. Nurses in this research reported the reasons behind the decision to emigrate, but also why they specifically decided to go to the United Kingdom.

### The choice of destination

There is evidence that colonial ties exert an influence on migration, particularly influencing the choice of destination [44,45,54]. For nurses from India this is the United Kingdom and for nurses from the Philippines, it is the United States. But sometimes nurses prefer other countries for different reasons, as with the Filipina nurses in this study. They cited contractual conditions, other than salary, as an important factor attracting them to the United Kingdom instead of the United States.

"Many of my friends are there [the United States] already, but I heard from them, that they only get two weeks' holidays and I think that it is more important for me to have longer holidays. During holidays I normally go home to visit my family and it's a long flight, 17 hours" (ID18 Filipina nurse with more than 46 months' experience in the United Kingdom and with no previous overseas experience).

Once the decision to emigrate is made, nurses start looking for job opportunities, often in a specific country but sometimes on the basis of available opportunities. The legal requirements for professional registration and immigration in the destination country and the complexity and length of these processes are also cited as influencing the choice of destination.

"No. 1 is USA but it is quite tough with applicants, you have to do so many tests, so many requirements, so many years of experience, before you qualify to the US, and even when you are already there you have to take examinations. I think United Kingdom is quite good, they only require you to do adaptation [programme], as long as you pass the adaptation, that's it...." (ID19 Filipina nurse with more than 55 months' experience in the United Kingdom and with no previous overseas experience).

Thus overall, nurses decided to emigrate pushed and pulled by economic and professional factors but also by the social and cultural environments in which they were living and by destination-country application requirements before deciding to move. The implications of these results are important, particularly in the context of the current global shortage of nurses [1].

## Discussion

As outlined above, many factors influence nurse migration. These findings validate other studies on internationally recruited nurses [24,25,27,29,30,38,40,55-59]. However, nurses in this study also identified the importance of family and friends regarding their decision to emigrate. The influence of the cultural environment has also been illustrated through the experiences of nurses working in Saudi Arabia before coming to the United Kingdom.

One important finding of this study is that the motivation of nurses coming to the United Kingdom for the first time differs from that of nurses coming to the United Kingdom after having worked in other migratory destinations. In analysing the factors involved in the decision to emigrate, we need to differentiate between the factors involved in the decision leading to the initial move undertaken by many of the nurses from their country of origin, most often to the Gulf States, and the factors involved in the decision to move from there to the United Kingdom.

All nurses in this research expected to improve their economic situation, but nurses coming from their countries of origin were more emphatic about these economic aspirations than those coming from previous migratory destinations such as Saudi Arabia. Using Maslow's hierarchy of needs theory, we suggest that an individual's behaviour is driven by those needs that are perceived to be the most important [60].

Nurses' perception of their economic needs changed after they had improved their financial situation in the first migratory destination; thereafter, professional, social or more personal factors became relatively stronger. In particular, those nurses who went to the Gulf states, for example to Saudi Arabia as a first migratory destination found that Saudi society was very restrictive for women, and the findings suggest this contributed to their decision to move again to the United Kingdom.

Initially economic needs were perceived as pre-eminent (as demonstrated in the first move), rather than the need for a more liberal social environment. Once economic needs were covered, social or personal reasons became more important. These findings corroborate Maslow's hypothesis. Having been attracted by different factors, nurses, with or without previous migratory experience, may develop different expectations and hence may need different stimuli to keep them satisfied.

Nurses coming from a first migratory destination, particularly those coming from the Gulf States, had already been exposed to a work environment that offered sophisticated technology and clinical resources. Some nurses who worked in the private health sector in their countries of origin were also used to well-resourced settings. In these cases, the experience in the United Kingdom largely did not meet their expectations.

By providing good information pre-departure about the resources that nurses can expect, employers in the United Kingdom could minimize any negative impact on motivation that unmet expectations may have in this regard, which may contribute to increased retention. Insufficient, inaccurate and sometimes misleading information received during the recruitment process can result in unmet expectations, causing a negative impact on their psychological contract with the employer, their motivation and potentially on their intention to stay or to leave.

An important element of the British nursing system, and one that attracts overseas nurses to work in the NHS, is its policy on professional development. The Nursing and Midwifery Council (NMC) Code of Professional Conduct states that as a registered nurse "...you must keep your knowledge and skills up-to-date throughout your working life. In particular, you should take part regularly in learning activities that develop your competence and performance" [61]. Due to the lifelong nature of professional development [62], nurses coming to the United Kingdom with the primary aspiration of improving their professional skills might pursue a longer-term engagement than those coming primarily for economic reasons.

It is difficult to assess the socioeconomic improvement nurses experienced by coming to the United Kingdom. There appear to be differences in this regard between the Indian and the Filipina nurses. Indian nurses, during the time covered by this study, were in the United Kingdom for too short a time to evaluate their improvements. However, they expressed dissatisfaction with living conditions, some observing a noticeable worsening in their living standards. Poor institutional accommodation; relatively low salaries when compared with nurses with similar experience but already registered with the NMC; low purchasing capacity due to the high cost of living in London; or meeting difficulties in bringing their families to the United Kingdom were all commonly reported problems.

Keeping families together often meant the husband had to leave his employment in India and look for a job in the United Kingdom. The family had to live on the nurse's salary until that happened, which made it difficult for them to achieve improvements in their socioeconomic status. Husbands often have to accept jobs below their previous positions in India.

The case of Filipina nurses was different. Most did not bring their family to the United Kingdom. They often lived in shared houses at a lower cost. Most of them mentioned that after one year they were attaining some of their economic goals, such as paying tuition fees for relatives or investing in businesses back home.

In studying how the decision to emigrate is made, it is important to examine the personal motivation of migrants and their families. Social and cultural influences are also involved, however. In the Philippines, migration is considered not only as a professional option, but one of survival. Almost half the population lives below the poverty line and unemployment represents an important problem. In this context, nursing is perceived as a good opportunity for life improvement. That doctors and other professionals in the Philippines are now undertaking nursing studies as an opportunity to emigrate supports this argument [6,9,63].

The postcolonial perspective cannot be underestimated when examining the reasons for migration [64]. Indian nurses came to the United Kingdom attracted by what they perceived as the source of their nursing education. Filipina nurses, for similar reasons, most often decide to migrate to the United States [54]. However, Filipina nurses in this study preferred the United Kingdom because it was easier to enter and attain registration as a nurse than in their traditional destination, the United States.

The Philippines represented the main source of overseas nurses to the United States in 2005 and the large flow of Filipina nurses attempting to migrate to the United States generates great competition. An average of 15 000 nurses from the Philippines take the tests required to work as a nurse in the United States; in 2005 only 42% of applicants passed them [65]. The number of visas issued by the United States Bureau of Citizenship and Immigration Services for nurses is limited.

For some nurses, such a competitive environment may have contributed to their decision to come to the United Kingdom, potentially as a first step, before moving to the United States, as reported by some of the nurses in this study. It was also suggested that the longer holidays offered in the United Kingdom was important for this group of nurses. Having their families in the Philippines, they wanted to go back regularly, requiring expensive, long-haul flights. This would be difficult if they were in the United States, where employers offer shorter holiday periods. Also the easier process of application and registration was mentioned as one of the positive aspects of the British system.

### Contributions, strengths and limitations of the study

This study has contributed to the existing body of knowledge about nurse migration. Exploration of factors other than the purely economic has illuminated areas such as the social and the cultural environment as important elements in the decision-making process that nurses undertake before leaving their home countries.

Identifying internationally recruited nurses as a homogeneous group may be misleading. This study has demonstrated the differences between the motivations of first-time migrants as compared with those reported by nurses with previous migratory experience, as well as the different motivations of Filipina and Indian nurses. The importance of the process of recruitment, particularly the information provided pre-departure, has been illustrated as affecting the psychological contract and thus influencing the commitment of nurses to the employer and their intention to stay or to leave. The study has also highlighted other aspects involved in the choice of destination, such as contractual conditions and regulatory aspects.

Some of the strengths of this study derive from its methodology, which allowed for a deep exploration of this relatively unknown phenomenon, giving voice to the nurses. Similar studies are based on focus groups, with nurses of relatively short migratory experience [29,40,66]. This study used individual semi-structured interviews that allowed nurses to provide accounts in a confidential environment and that would have been unlikely to have been obtained through group interviews.

The comparative nature of this research represents an important methodological contribution in the existing nurse migration literature. First, the longitudinal element of the research and that it involved nurses with different lengths of experience in the United Kingdom – from one day to more than four years – add to the strengths of this study. Then, studying nurses coming to the United Kingdom from only two countries has allowed us to highlight conclusions such as the cultural specificity of nurses coming from different countries and cultures that could not have been reached if the participants were of more varied origins.

Some potential limitations of the study are derived from the fact that the recruitment of participants was undertaken by the Recruitment and Retention Department in the Trust. Selection may have been biased by the organization's interests, but the fact that the authors provided selection criteria may have minimized any bias.

Another potential constraint is the gender bias introduced during the recruitment of participants. All participants were women. None of the nurses recruited in India and only a few of those recruited in the Philippines were men however; thus the subsample reflects the total population. It can be argued that one limitation of case studies is the generalizability of their findings. However, that was not the objective of the study and the theories constructed from the findings can be tested in other similar contexts such as those studied by Allan and Larsen (2003) or Smith et al. (2006) [24,30].

## Conclusion

Nurses' decision to emigrate is complex and not based solely on economic expectations. By analysing the reasons nurses from India and the Philippines reported for leaving their countries and those that attracted them to the United Kingdom, this study has provided more evidence about factors important to nurses that in the hands of policy-makers can help health systems in developing countries to increase retention of this essential and often scarce resource.

Internationally recruited nurses cannot be seen as one group. Nurses from different countries or with different migration experience have different expectations and therefore their motivation varies. Whilst this study validates the results of other studies, in which economic factors were seen as a contributor to the final decision, it identifies other factors that in some circumstances outweigh the financial aspects of migration. The implication is that economic incentives may be just part of the solution, but improving working environments, providing minimum material and equipment, offering professional development opportunities and improving supervision may prove just as important for increasing retention of greatly needed human resources. The study found that the social and cultural environments where nurses live have a great influence in the decision to emigrate.

By helping to understand the expectations of nurses who move to the United Kingdom, the findings of this study also help in defining the terms of the psychological contract [19] that nurses develop with the United Kingdom employer, which, if taken into account, can improve the experience of Indian and Filipino nurses recruited internationally and therefore increase their retention. This may help reduce NHS recruitment of nurses from other countries where they are greatly needed.

## Competing interests

The authors declare that they have no competing interests.

## Authors' contributions

AA-G conceived and designed the study and collected the data. Both authors contributed to the analysis and interpretation of data. AA-G drafted the manuscript and JM critically revised it with substantial intellectual contribution.
